# Supplementary material for: Chemical Characterization, Antioxidant, Cytotoxic and Microbiological Activities of the Essential Oil of Leaf of Tithonia Diversifolia (Hemsl) A. Gray (Asteraceae)
Source: Pharmaceuticals (Basel). 2019 Mar 4;12(1):34. doi: 10.3390/ph12010034 (PMC6469183; doi:10.3390/ph12010034)
Supplement: Supplementary file 1 [file pharmaceuticals-12-00034-s001.pdf]

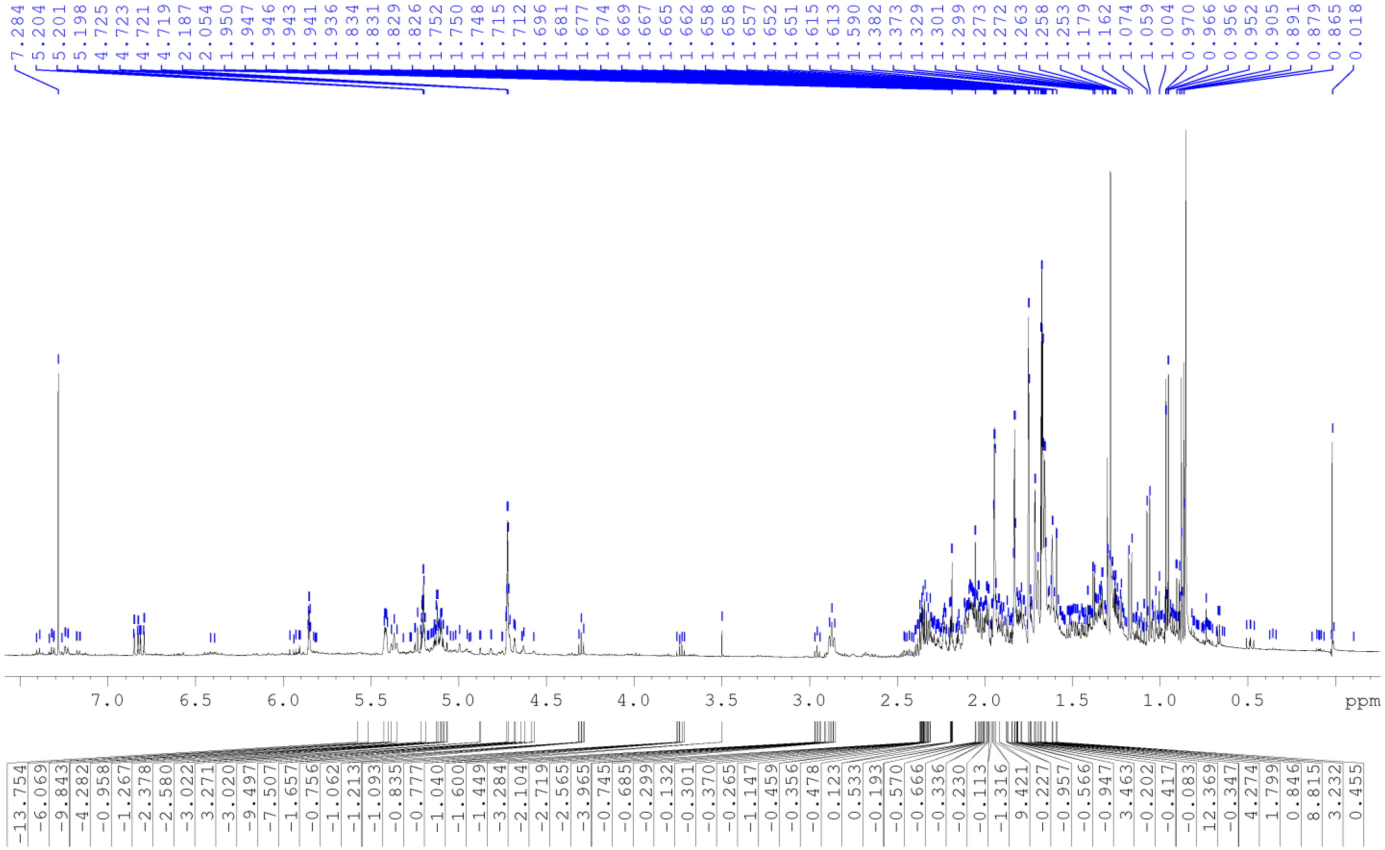

Figure S1. The ^{1}H NMR spectrum ($CDCl_3$, 400 MHz) of the essential oil of the leaves of *T. diversifolia*.

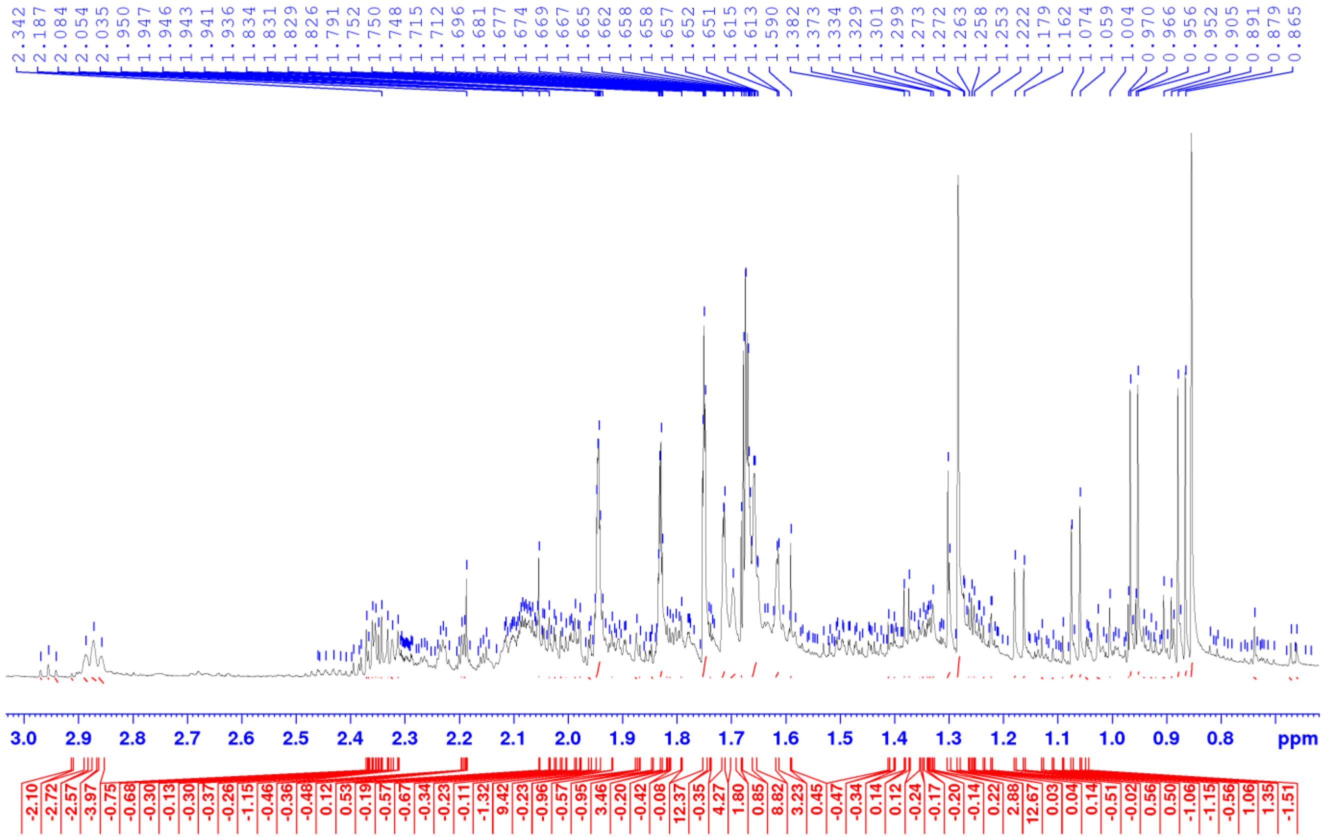

Figure S2. Amplification (0.5–3.0) of the ^{1}H NMR spectrum ($CDCl_3$, 400 MHz) of the essential oil of the leaves of *T. diversifolia*.

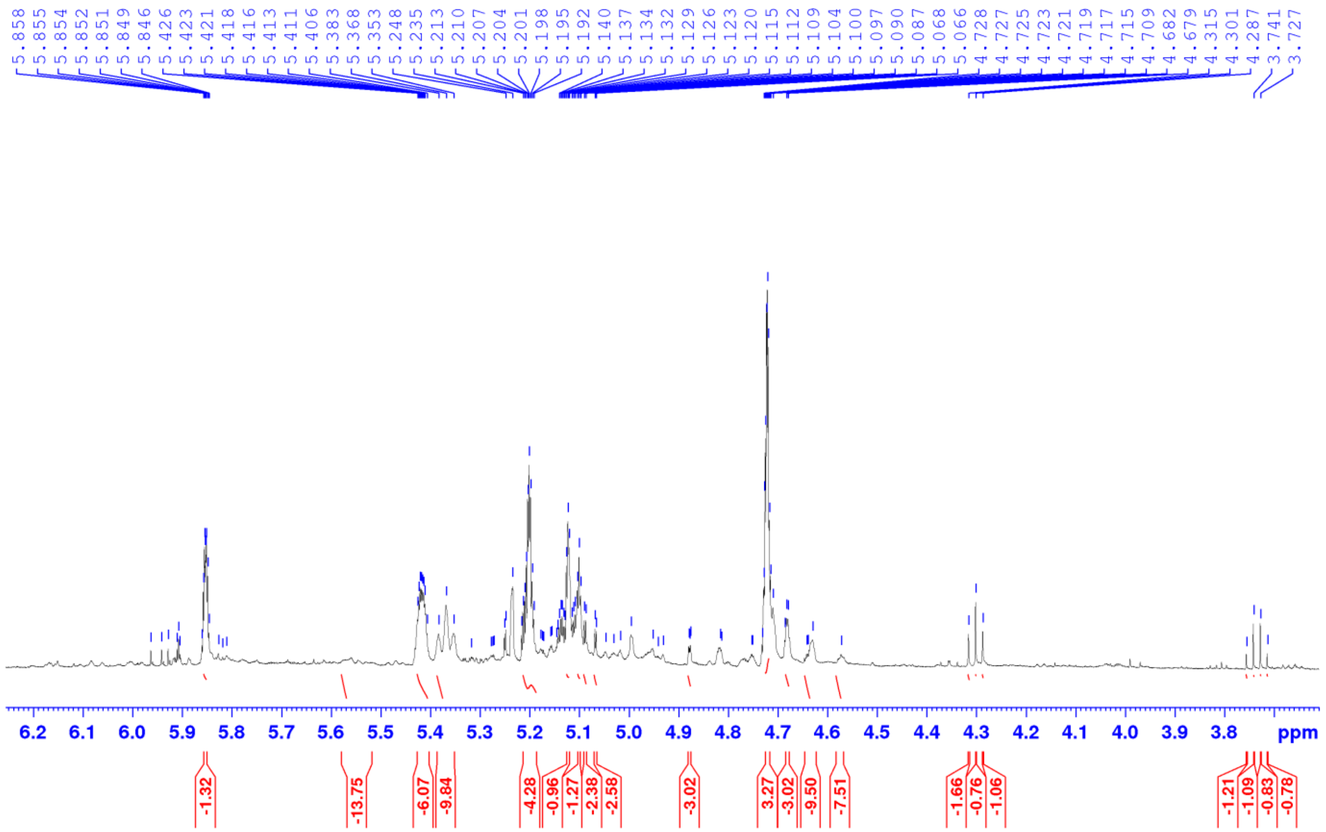

Figure S3. Amplification (6.2–3.8) of the ^{1}H NMR spectrum ($CDCl_3$, 400 MHz) of the essential oil of the leaves of *T. diversifolia*.

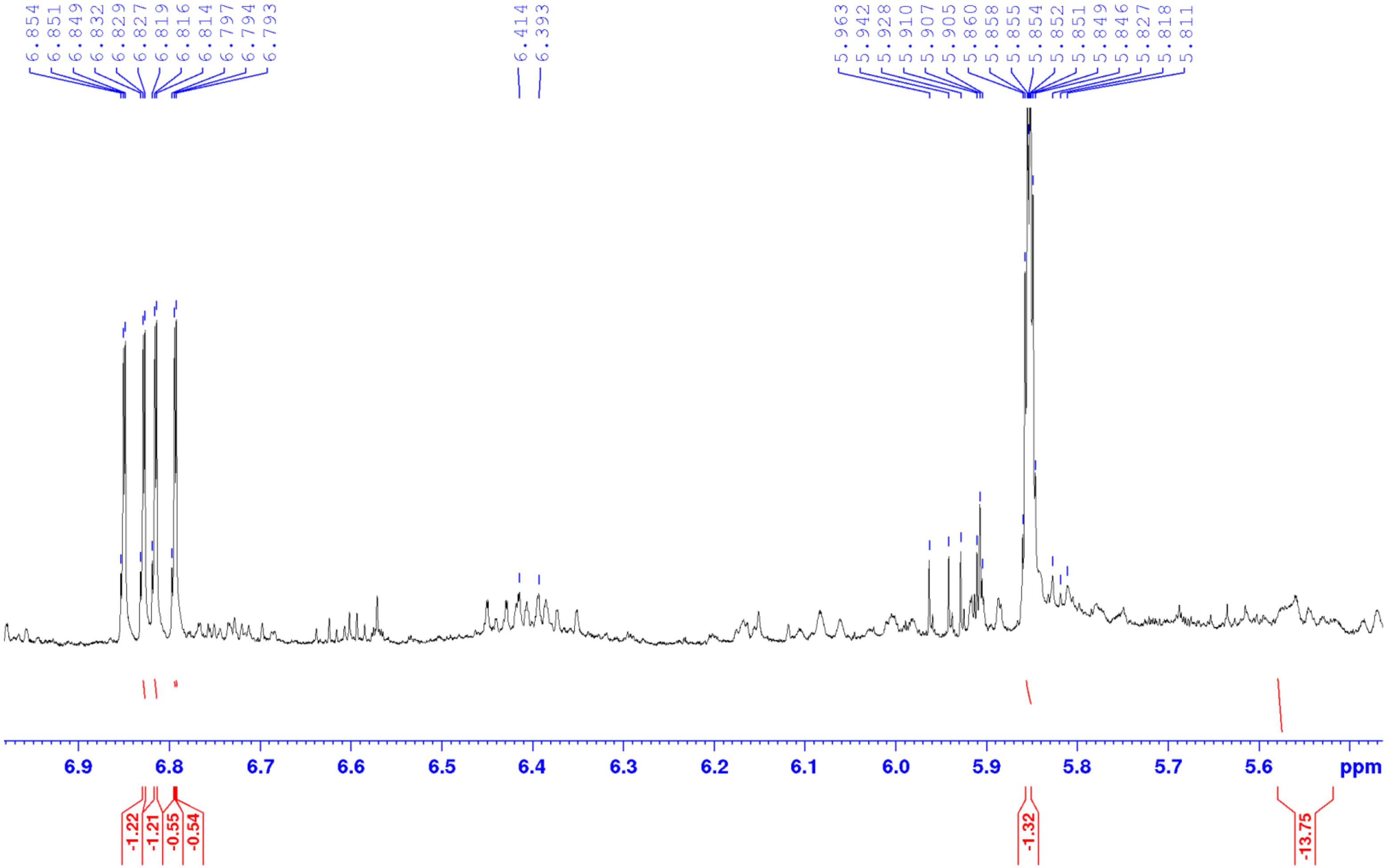

Figure S4. Amplification (6.9–5.6) of the ^{1}H NMR spectrum ($CDCl_3$, 400 MHz) of the essential oil of the leaves of *T. diversifolia*.

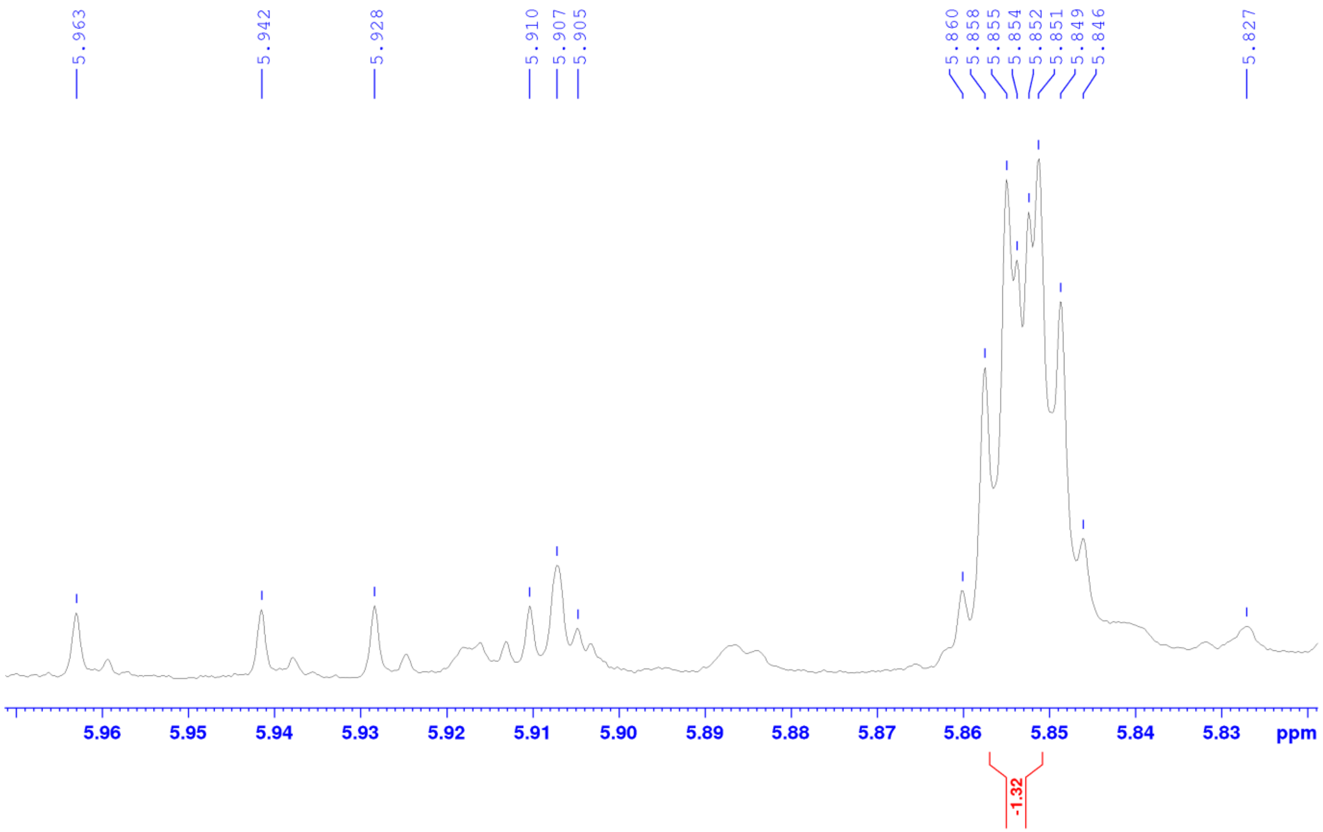

Figure S5. Amplification (5.96–5.83) of the ^{1}H NMR spectrum ($CDCl_3$, 400 MHz) of the essential oil of the leaves of *T. diversifolia.*

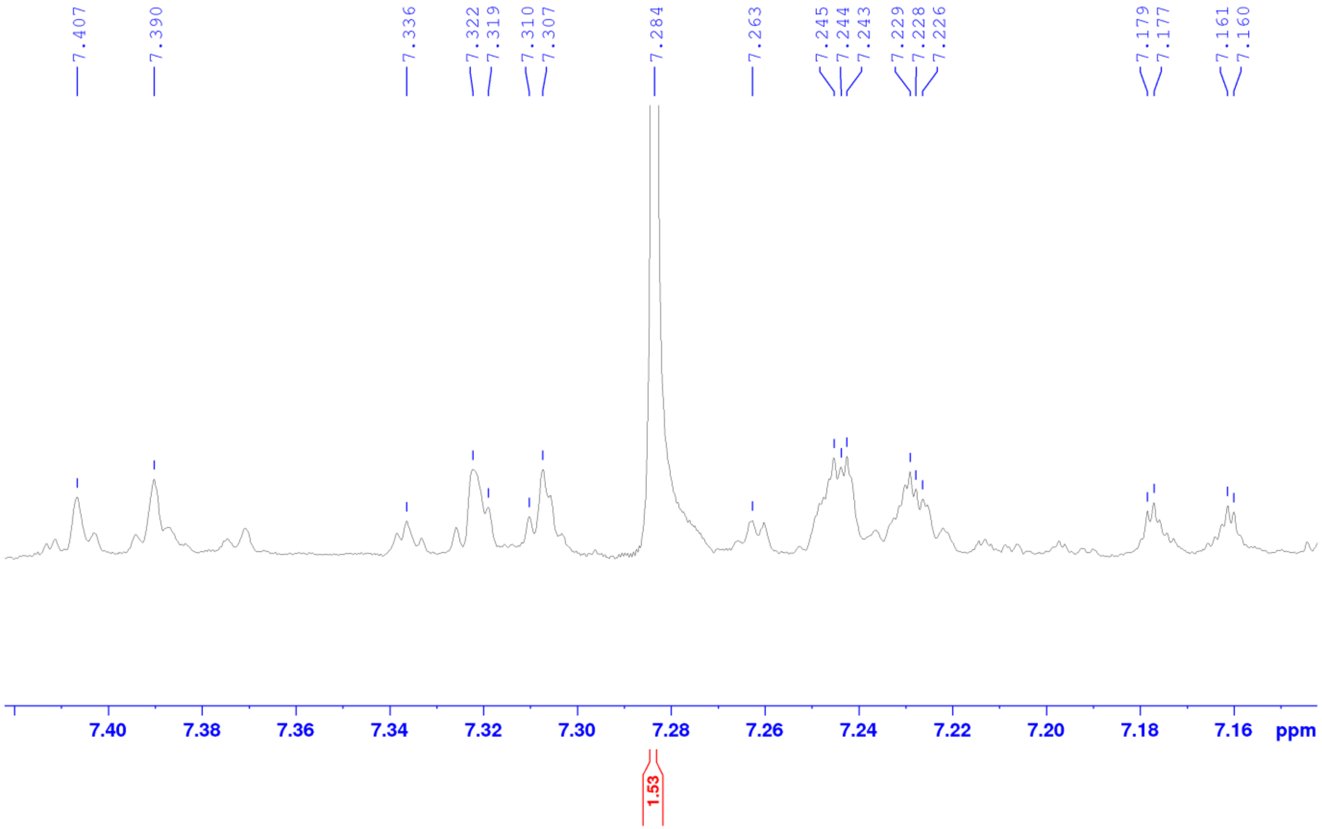

Figure S6. Amplification (7.40–7.16) of the ^{1}H NMR spectrum ($CDCl_3$, 400 MHz) of the essential oil of the leaves of *T. diversifolia.*

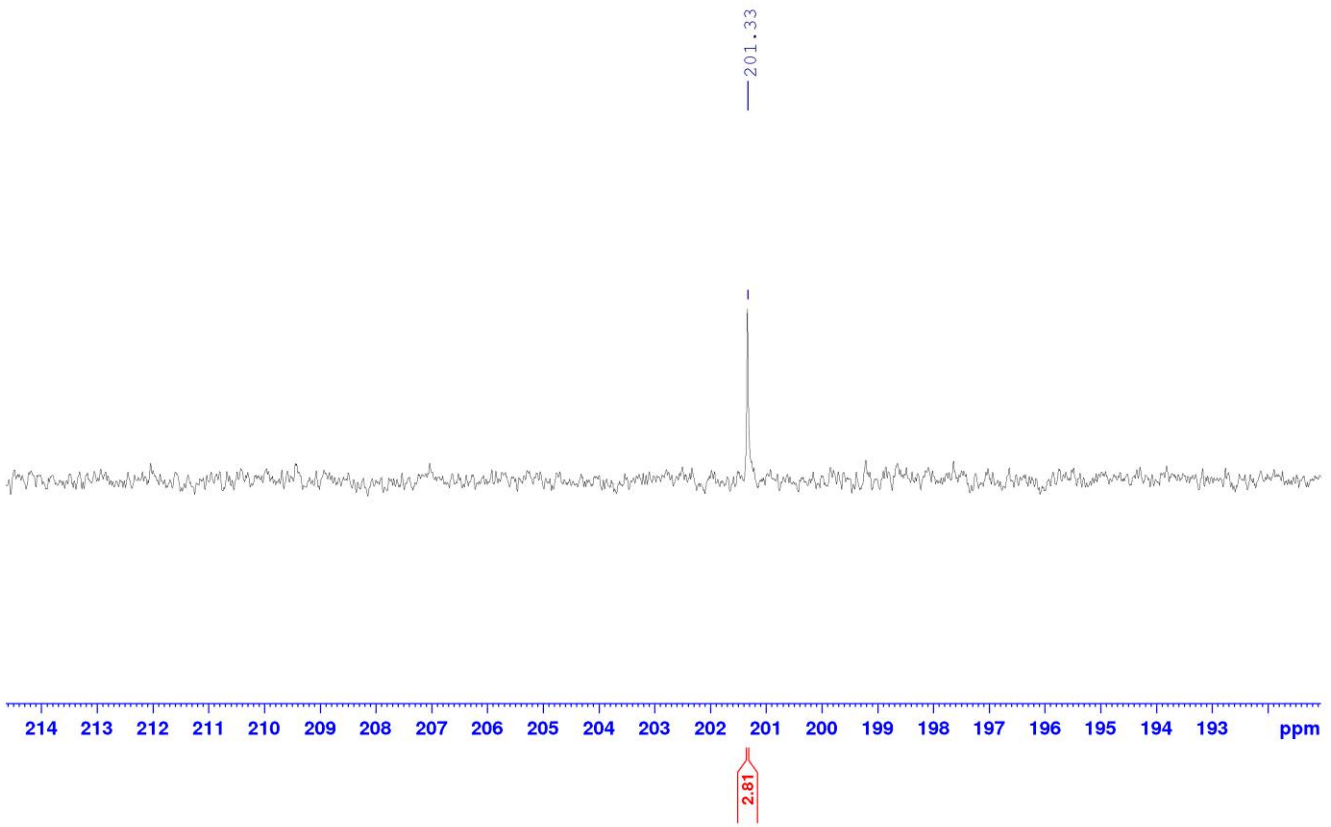

Figure S7. Amplification (214–193) of the ^{13}C NMR spectrum ($CDCl_3$, 400 MHz) of the essential oil of the leaves of *T. diversifolia.*

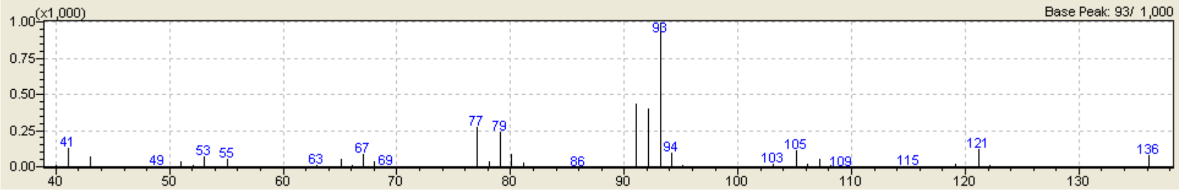

Compound mass spectrum α-pinene.

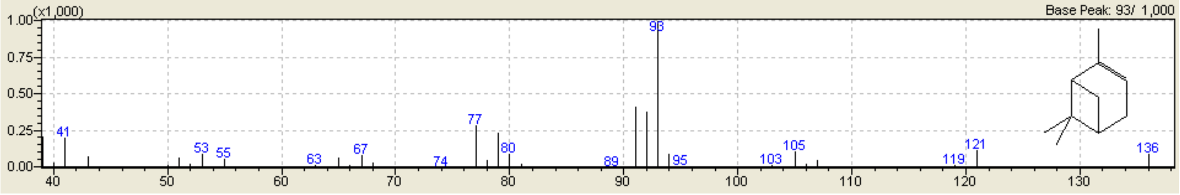

Spectrum of substance substance α-pinene of the equipment NIST05 and WILEY'S Library of Mass spectra 9th Edition.

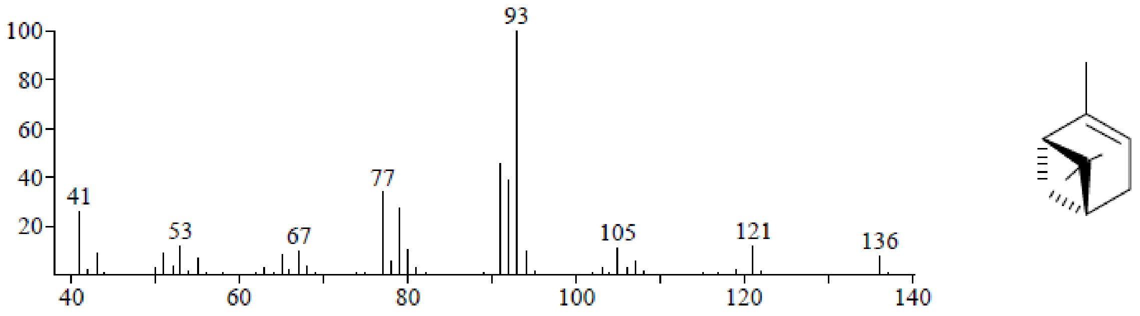

Literature mass spectrum (Adams, 2017).

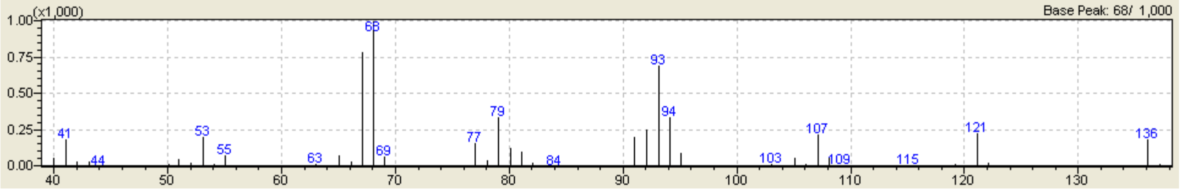

Compound mass spectrum Limonene.

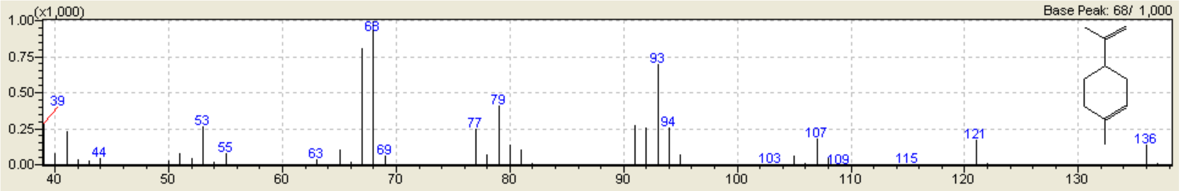

Spectrum of substance substance Limonene of the equipment NIST05 and WILEY'S Library of Mass spectra 9th Edition.

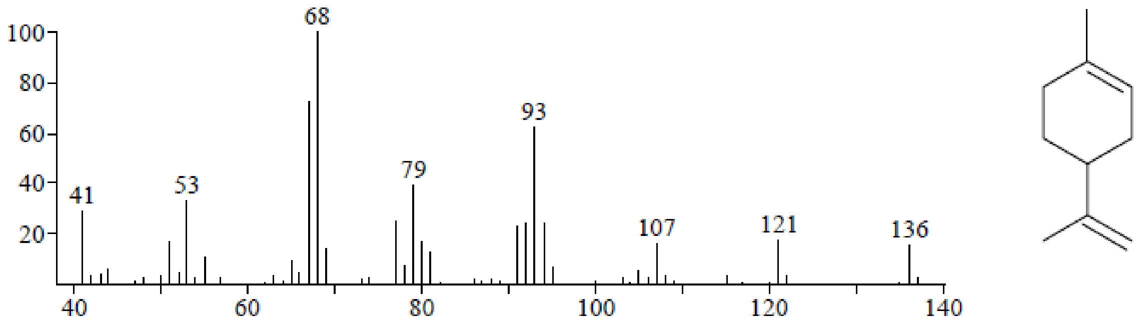

Literature mass spectrum (Adams, 2017).

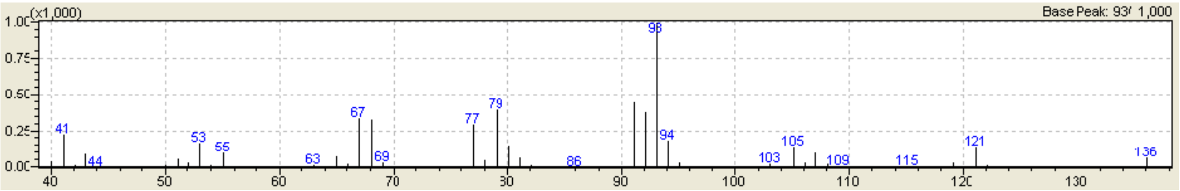

Compound mass spectrum *Z*-*β*-ocimene.

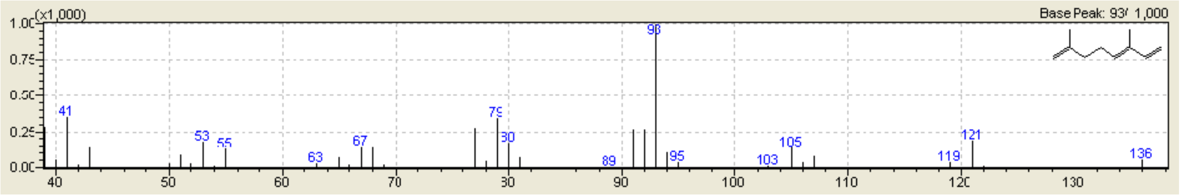

Spectrum of substance substance *Z*-*β*-ocimene of the equipment NIST05 and WILEY'S Library of Mass spectra 9th Edition.

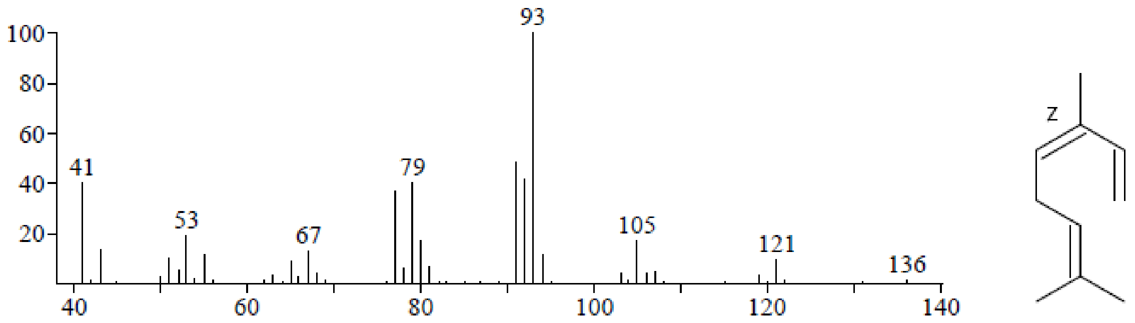

Literature mass spectrum (Adams, 2017).

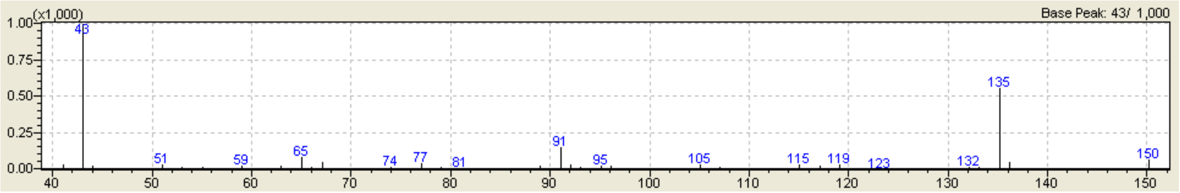

Compound mass spectrum *p*-cymen-8-ol.

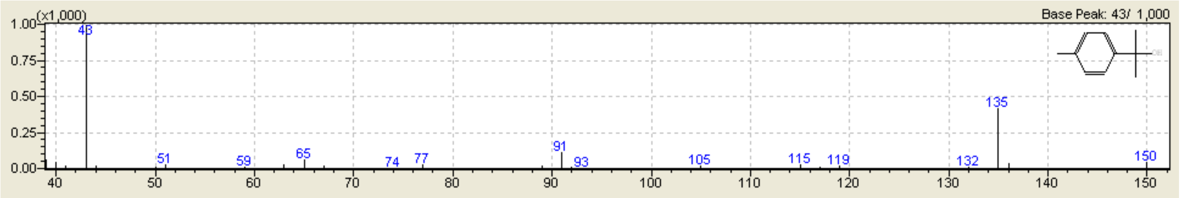

Spectrum of substance substance *p*-cymen-8-ol of the equipment NIST05 and WILEY'S Library of Mass spectra 9th Edition.

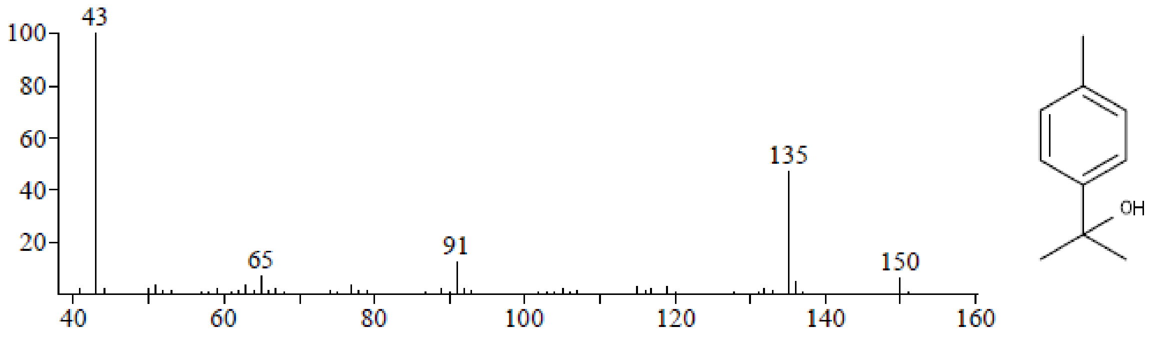

Literature mass spectrum (Adams, 2017).

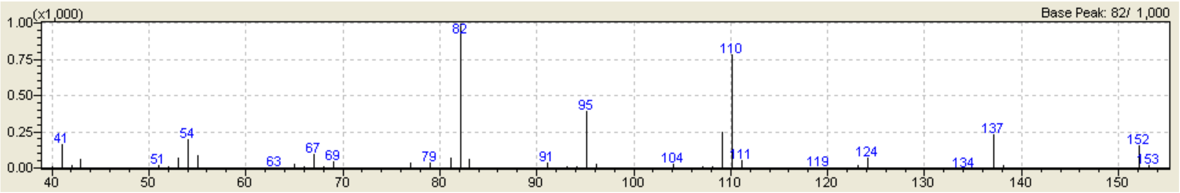

Compound mass spectrum Piperitone.

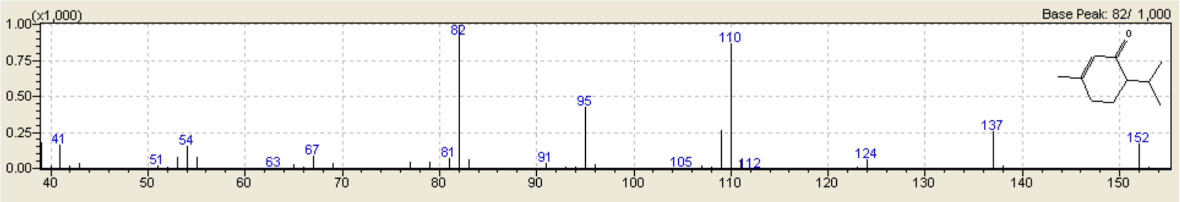

Spectrum of substance substance Piperitone of the equipment NIST05 and WILEY'S Library of Mass spectra 9th Edition.

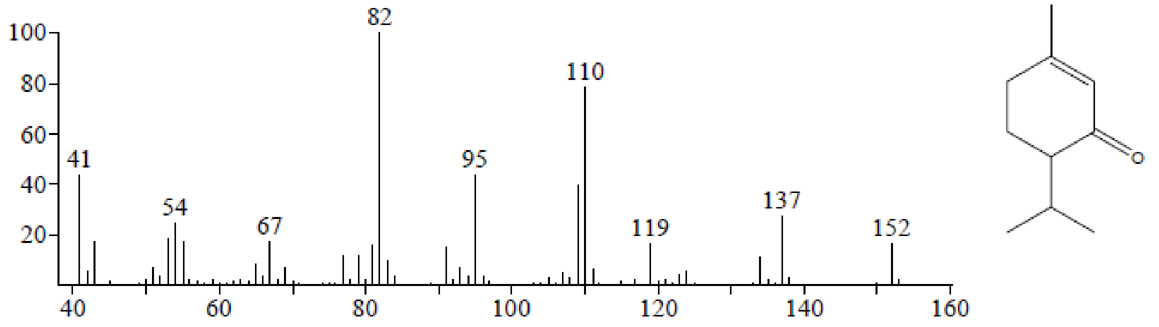

Literature mass spectrum (Adams, 2017).

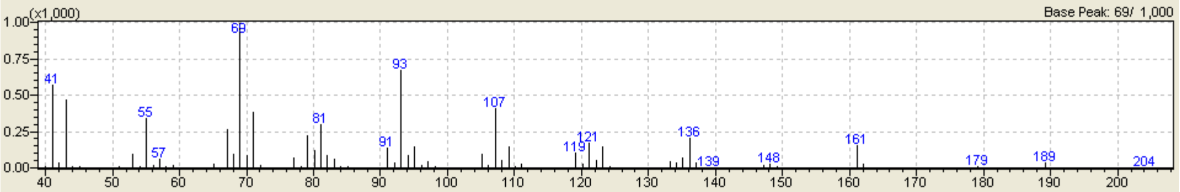

Compound mass spectrum *E*-*n*erolidol.

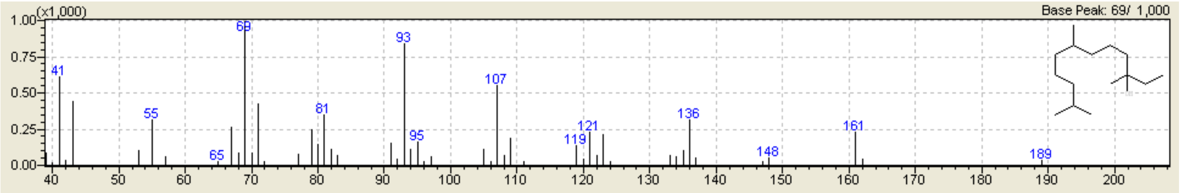

Spectrum of substance substance *E*-*n*erolidol of the equipment NIST05 and WILEY'S Library of Mass spectra 9th Edition.

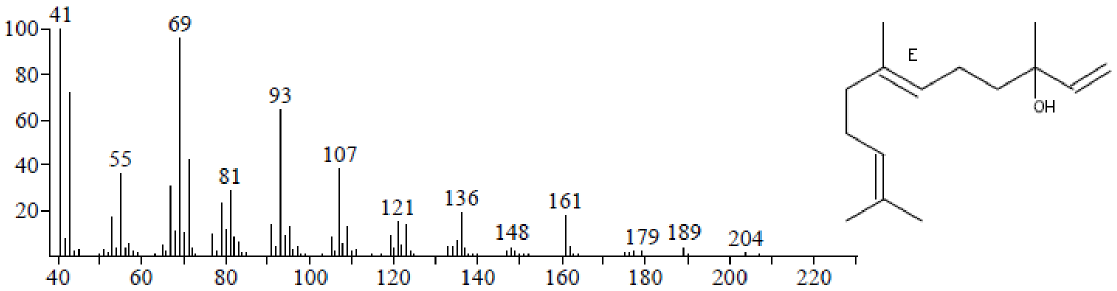

Literature mass spectrum (Adams, 2017).

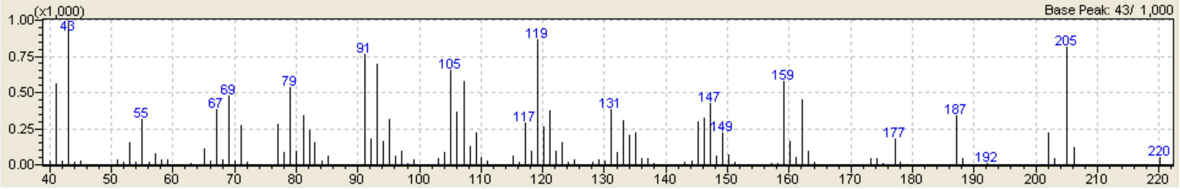

Compound mass spectrum Spathulenol.

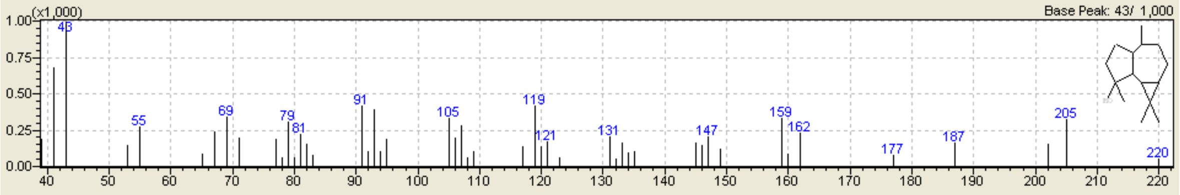

Spectrum of substance substance Spathulenol of the equipment NIST05 and WILEY'S Library of Mass spectra 9th Edition.

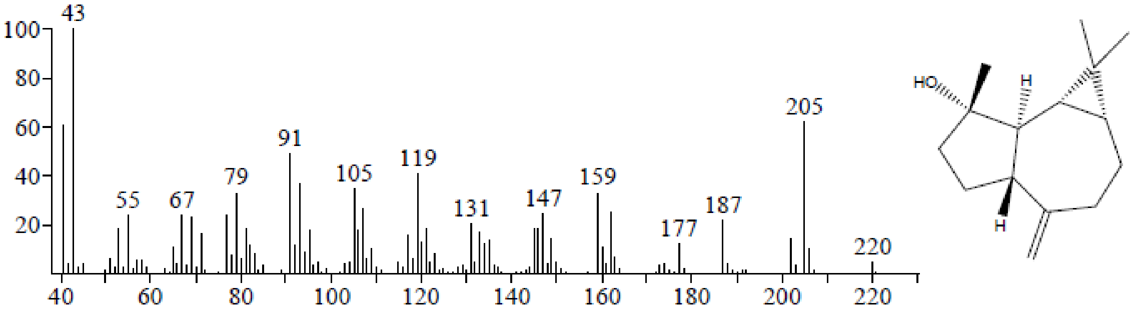

Literature mass spectrum (Adams, 2017).

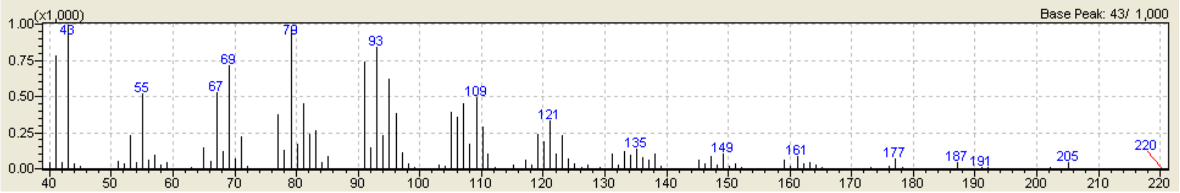

Compound mass spectrum Caryophyllene oxide.

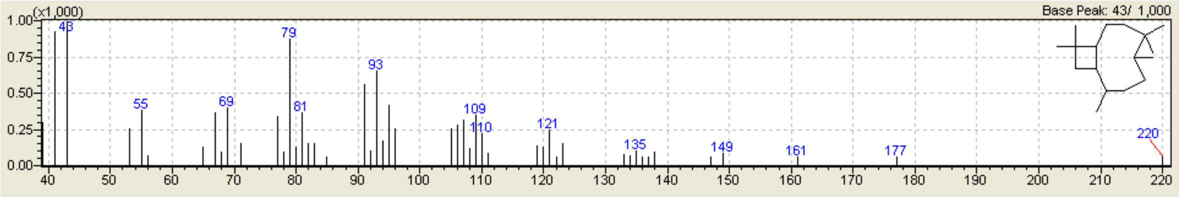

Spectrum of substance substance Caryophyllene oxide of the equipment NIST05 and WILEY'S Library of Mass spectra 9th Edition.

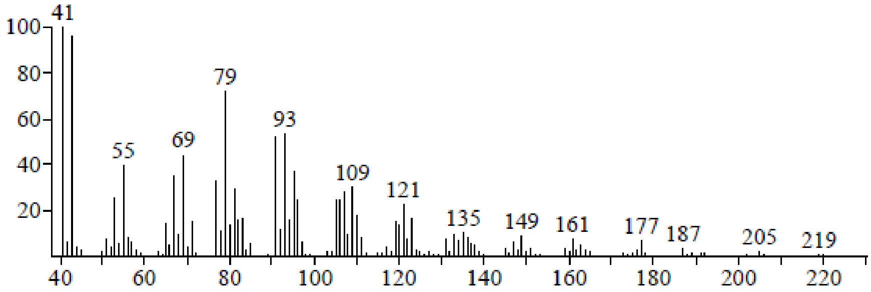

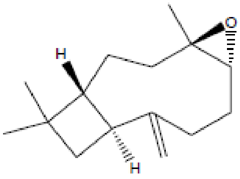

Literature mass spectrum (Adams, 2017).

Figure S8. Mass spectra of the major compounds *Tithonia diversifolia* and ^{1}H NMR spectrum and ^{13}C essential oil of *T. diversifolia*.
